# Development and external validation of a pretrained deep learning model for the prediction of non-accidental trauma

**DOI:** 10.1038/s41746-023-00875-y

**Published:** 2023-07-19

**Authors:** David Huang, Steven Cogill, Renee Y. Hsia, Samuel Yang, David Kim

**Affiliations:** 1 Department of Computer Science, Stanford University, Stanford, CA USA; 2 Department of Veterans Affairs, Seattle, WA USA; 3 Department of Emergency Medicine, UCSF School of Medicine, San Francisco, CA USA; 4 Department of Emergency Medicine, Stanford University, Stanford, CA USA

**Keywords:** Predictive medicine, Preventive medicine, Trauma

## Abstract

Non-accidental trauma (NAT) is deadly and difficult to predict. Transformer models pretrained on large datasets have recently produced state of the art performance on diverse prediction tasks, but the optimal pretraining strategies for diagnostic predictions are not known. Here we report the development and external validation of Pretrained and Adapted BERT for Longitudinal Outcomes (PABLO), a transformer-based deep learning model with multitask clinical pretraining, to identify patients who will receive a diagnosis of NAT in the next year. We develop a clinical interface to visualize patient trajectories, model predictions, and individual risk factors. In two comprehensive statewide databases, approximately 1% of patients experience NAT within one year of prediction. PABLO predicts NAT events with area under the receiver operating characteristic curve (AUROC) of 0.844 (95% CI 0.838–0.851) in the California test set, and 0.849 (95% CI 0.846–0.851) on external validation in Florida, outperforming comparator models. Multitask pretraining significantly improves model performance. Attribution analysis shows substance use, psychiatric, and injury diagnoses, in the context of age and racial demographics, as influential predictors of NAT. As a clinical decision support system, PABLO can identify high-risk patients and patient-specific risk factors, which can be used to target secondary screening and preventive interventions at the point-of-care.

## Introduction

Non-accidental trauma (NAT) comprises a heterogeneous set of diagnoses, including many forms of assault, abuse, maltreatment, and neglect. NAT is a leading cause of injury and death, particularly for children and adolescents^1,2^, pregnant and postpartum women^3,4^, and disadvantaged social groups^5,6^. NAT is also difficult to predict, due to heterogeneity of presentation, complex and rapidly changing epidemiology^7^, and concentration in historically understudied populations^8^. Screening for NAT during clinical encounters is not routine, and may be associated with both provider and patient discomfort^9^. Therefore, the effects of routine screening for NAT have not been well-demonstrated, except in special populations^10^.

An automated screening algorithm using existing electronic health record (EHR) data could enable universal screening for high-risk patients without requiring additional clinical resources or imposing a social burden. A limited number of studies have evaluated the predictability of NAT. Most research has been restricted to identification of broad risk factors, such as psychiatric illness and prior involvement in violence^11,12^. Prior research has also associated NAT with specific clinical contexts, as in children with fracture patterns suggestive of NAT^13^. One previous modeling study evaluated a Bayesian classifier for predicting a variety of NAT diagnoses^14^. This approach used a flattened representation of patient histories, precluding use of information about the sequence and tempo of visits. More generally, prior studies of NAT prediction have not shown external validation^15^, an important assessment of the ability of a model to generalize to different patient populations and clinical environments.

Deep learning models based on transformer architectures^16^ have achieved state-of-the-art performance in multiple domains^17,18^, including disease prognostication^19,20^. Bidirectional Encoder Representations from Transformers^21^ (BERT) is a popular architecture that can effectively learn long-range patterns from sequence data, making it promising for the prediction of longitudinal outcomes. Such models benefit from general pretraining on massive datasets, which improves performance when the model is subsequently fine-tuned on a specific prediction task^21^. However, previous studies of disease prediction have focused primarily on generic pretraining methods such as masked language modeling (MLM)^19^ and contrastive learning^22^, which are not specifically tailored to the characteristics of medical trajectories. One study used a domain-adapted pretraining task for predicting prolonged length-of-stay^20^, but not specific diagnoses or outcomes.

Our goal is to develop a clinically relevant prediction framework for NAT and other challenging diagnoses, using longitudinal patient trajectories containing patient demographics, diagnoses, and procedures. Using statewide data on millions of encounters, we pretrain a BERT-based model adapted for longitudinal diagnostic prediction with a multitask pretraining objective. By flexibly predicting the temporal relationships of diagnoses, we aim to develop a generally applicable base model for diagnostic forecasting. We fine-tune and externally validate our model for the prediction of NAT. We compare model performance to a traditional machine learning algorithm and a previously published BERT-based model. We develop an interactive clinical interface for understanding model predictions and individual risk factors, which can be implemented as a clinical decision support system.

## Results

### Study overview

**Fig. 1:**
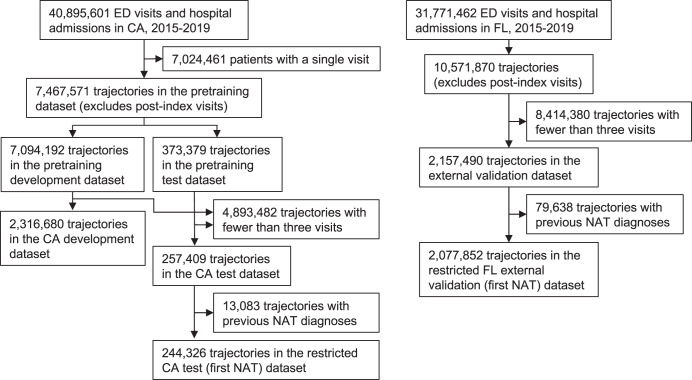
Cohort creation. Our CA dataset (left) was divided into development and test splits at a 95:5 ratio for pretraining and a 9:1 ratio for finetuning using random sampling at the patient level. For pretraining, we included trajectories with two or more visits. For finetuning, we included trajectories with three or more visits. We also created a CA test dataset for “first NAT” that excluded trajectories with previous NAT diagnoses. Our FL external validation datasets (right) were created with the same inclusion and exclusion criteria as the CA test datasets. CA = California. FL = Florida. ED = Emergency Department. NAT = non-accidental trauma.

**Fig. 2:**
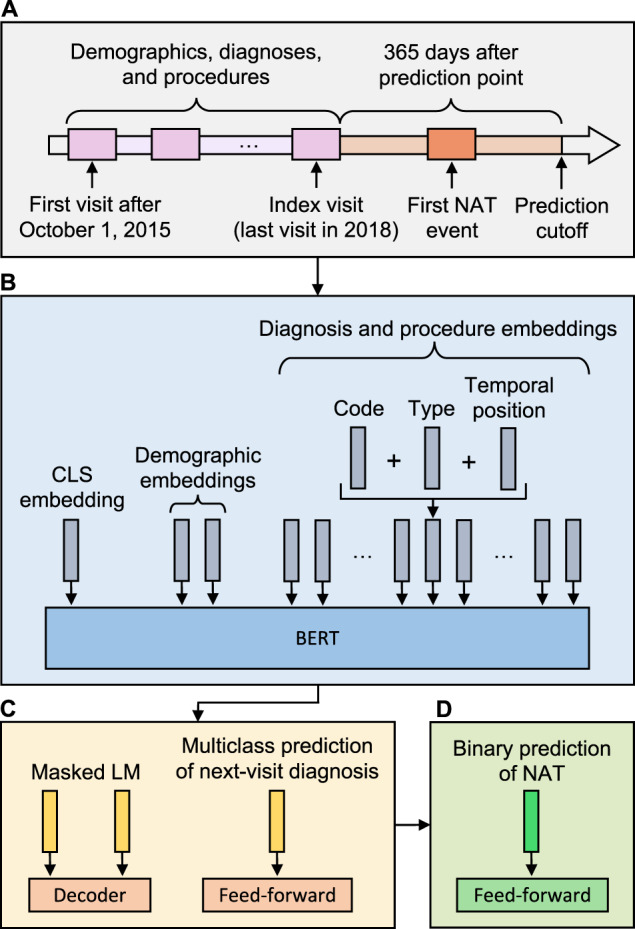
Modeling approach. **A** Visit records from October 1, 2015 to the patient’s last visit in 2018 (the index visit) were used to predict non-accidental trauma (NAT) within one year of the index visit. **B** PABLO embedding schema. Dense embeddings for codes, code types (i.e., principal or auxiliary), and temporal positions were summed to produce a single representation for each feature. A summary classification (CLS) token was used for prediction. **C** Multitask pretraining. In addition to predicting masked features, we included a multiclass objective for predicting the diagnosis category of the patient’s next visit. **D** Fine-tuning PABLO for binary prediction of NAT within 365 days of the index visit. PABLO = Pretrained and Adapted BERT for Longitudinal Outcomes.

We developed Pretrained and Adapted BERT for Longitudinal Outcomes (PABLO), which we fine-tuned and validated for the prediction of NAT within one year. Cohort creation for pretraining, development, test, and external validation datasets is shown in Fig. 1. Cohort characteristics are reported in Table 1. Figure 2 summarizes the modeling approach, which encodes the longitudinal structure of patients’ medical trajectories.

**Table 1:** Cohort characteristics.

	CA development	CA test	FL external validation
Trajectories	2,316,680	257,409	2,157,490
Age, median (IQR)	48 (29–67)	48 (29–67)	45 (28–66)
Sex			
Female, *n* (%)	1,364,577 (59%)	151,519 (59%)	1,306,784 (61%)
Male, *n* (%)	952,103 (41%)	105,890 (41%)	850,706 (39%)
Race/ethnicity			
White, *n* (%)	992,091 (43%)	110,426 (43%)	1,142,171 (53%)
Black, *n* (%)	310,767 (13%)	34,563 (13%)	566,716 (26%)
Hispanic, *n* (%)	835,839 (36%)	92,588 (36%)	423,858 (20%)
Asian, *n* (%)	139,109 (6%)	15,522 (6%)	11,707 (1%)
Native, *n* (%)	12,417 (1%)	1383 (1%)	2168 (0%)
Other, *n* (%)	26,457 (1%)	2927 (1%)	10,870 (1%)
Payor			
Medicare, *n* (%)	738,292 (32%)	81,569 (32%)	685,479 (32%)
Medicaid, *n* (%)	927,476 (40%)	103,513 (40%)	537,357 (25%)
Private, *n* (%)	491,640 (21%)	54,743 (21%)	480,549 (22%)
Self, *n* (%)	97,026 (4%)	10,790 (4%)	328,502 (15%)
Other, *n* (%)	62,246 (3%)	6794 (3%)	125,603 (6%)
Number of visits, median (IQR)	5 (4–8)	5 (4-8)	6 (4–9)
Codes per visit, median (IQR)	7 (4–11)	7 (4-11)	9 (6–13)
Number of facilities	446	443	217
NAT prevalence, *n* (%)	28,370 (1%)	3140 (1%)	18,364 (1%)

*CA* California, *FL* Florida, *NAT* non-accidental trauma.

### Prediction of non-accidental trauma

In each cohort, 1% of patients experienced NAT within one year of prediction (Table 1). PABLO predicted NAT with the best area under the receiver-operating characteristic curve (AUROC) and area under the precision-recall curve (AUPRC) of the models we compared across all evaluation datasets (Table 2). On the California (CA) test dataset, PABLO achieved an AUROC of 0.844 (95% CI 0.838–0.851) and AUPRC of 9.45 × 10^−2^ (95% CI 8.70 × 10^−2^ − 10.32 × 10^−2^). For prediction of first NAT (i.e., excluding patients with NAT diagnoses prior to the index visit), PABLO had an AUROC of 0.820 (95% CI 0.812–0.828) and AUPRC of 4.80 × 10^−2^ (4.38 × 10^−2^ − 5.37 × 10^−2^). ROC curves are shown in Supplementary Fig. 1, and test characteristics at several operating points in Supplementary Table 1.

**Table 2:** Comparison of model performance in predicting non-accidental trauma.

Cohort	Trajectories	Logistic Regression		XGBoost		BEHRT		PABLO	
		AUROC	AUPRC	AUROC	AUPRC	AUROC	AUPRC	AUROC	AUPRC
CA test: any NAT	257,409	0.782 (0.774–0.789)	4.50 × 10^−2^ (4.23–4.81)	0.828 (0.820–0.834)	8.96 × 10^−2^ (8.20–9.83)	0.834 (0.827–0.841)	9.31 × 10^−^^2^ (8.54–10.19)	0.844 *†§ (0.838–0.851)	9.45 × 10^−^^2^ *† (8.70–10.32)
CA test: first NAT	244,326	0.762 (0.753–0.771)	2.91 × 10^−^^2^ (2.71–3.20)	0.798 (0.789–0.806)	4.15 × 10^−2^ (3.80–4.63)	0.806 (0.797–0.814)	4.46 × 10^−^^2^ (4.06–4.98)	0.820 *†§ (0.812–0.828)	4.80 × 10^−2^ *†§ (4.38–5.37)
FL external validation: any NAT	2,157,490	0.780 (0.777–0.783)	3.03 × 10^−2^ (2.95–3.12)	0.830 (0.827–0.833)	6.24 × 10^−2^ (6.00–6.50)	0.843 (0.840–0.846)	6.34 × 10^−2^ (6.11–6.57)	0.849 *†§ (0.846–0.851)	6.78 × 10^−2^ *†§ (6.54–7.05)
FL external validation: first NAT	2,077,852	0.765 (0.762–0.769)	2.10 × 10^−^^2^ (2.04–2.17)	0.807 (0.803–0.810)	3.22 × 10^−2^ (3.11–3.36)	0.823 (0.820–0.826)	3.54 × 10^−^^2^ (3.41–3.68)	0.830 *†§ (0.827–0.833)	3.64 × 10^−2^ *†§ (3.51–3.79)

Ranges in parentheses are 95% confidence intervals. For both CA and FL, we evaluated prediction of both first diagnoses of non-accidental trauma (“first NAT”) as well as any NAT diagnosis, including recurrent diagnoses (“any NAT”). *CA* California, *FL* Florida, *AUROC* area under the receiver operating characteristic curve, *AUPRC* area under the precision-recall curve. * = *p* < 0.05 improvement over logistic regression, † = *p* < 0.05 improvement over XGBoost, § = *p* < 0.05 improvement over BEHRT. Confidence intervals and model comparisons calculated via bootstrapping with 10,000 resamples.

Prediction performance in external validation was very similar, with AUROC of 0.849 (95% CI 0.846–0.851) and AUPRC of 6.78 × 10^−^^2^ (95% CI 6.54 × 10^−^^2^ − 7.05 × 10^−2^). In external validation of first NAT prediction, PABLO had an AUROC of 0.830 (95% CI 0.827–0.833) and AUPRC of 3.64 × 10^−^^2^ (3.51 × 10^−2^ − 3.79 × 10^−2^). ROC curves for external validation are shown in Supplementary Fig. 1, and test characteristics at several operating points in Supplementary Table 2. Across all prediction tasks (any or first NAT, in CA or FL), PABLO’s AUROC was significantly greater than comparator models (logistic regression, XGBoost, BEHRT) at *p* < 0.05, evaluated via bootstrapping with 10,000 resamples (Table 2). PABLO’s AUPRC was likewise significantly greater than comparator models, except for prediction of any NAT in the CA test set, where PABLO’s AUPRC improvement over BEHRT was not statistically significant.

Supplementary Table 3 shows performance metrics for PABLO and comparator models, at operating points set to classify the 1% of visits with highest predicted risk as potential NAT. Positive predictive value (PPV) reflects the extreme class imbalance in predicting NAT (only 1% of patients experience the outcome within the one-year prediction horizon). In external validation, PABLO had PPV of 0.121 (95% CI, 0.117–0.126), compared to 0.061 for logistic regression (95% CI, 0.058–0.064), 0.113 (95% CI, 0.109–0.117) for XGBoost, and 0.116 for BEHRT (95% CI, 0.112–0.120). In pairwise tests for PPV differences using bootstrap resampling, PABLO’s PPV outperformed each comparator model at *p* < 0.05. Flagging the same proportion of visits, PABLO would detect 98.4% more cases of actual NAT than logistic regression, 7.1% more cases than XGBoost, and 4.3% more cases than BEHRT.

We evaluated prediction performance on demographic subgroups (Supplementary Tables 4–5). Predictions were more accurate for patients aged 40 years or more at the index visit, and were somewhat less accurate for Black patients compared to other racial groups. Predictions were more accurate for patients with more visits, who also had higher prevalence of NAT. PABLO significantly outperformed XGBoost in almost all subgroups, and in many cases demonstrated less between-group variation in prediction performance.

### Effect of pretraining on prediction performance

We compared the performance of PABLO to an analogous model without pretraining at various training set sizes (Supplementary Fig. 2). Pretraining significantly improved prediction performance, particularly with less training data. With 1000 training examples, for instance, pretraining improved AUROC by 9% and AUPRC by 48% on the California test set. Pretraining also substantially improved PABLO’s performance in external validation, suggesting improved generalization.

### Feature representations

**Fig. 3:**
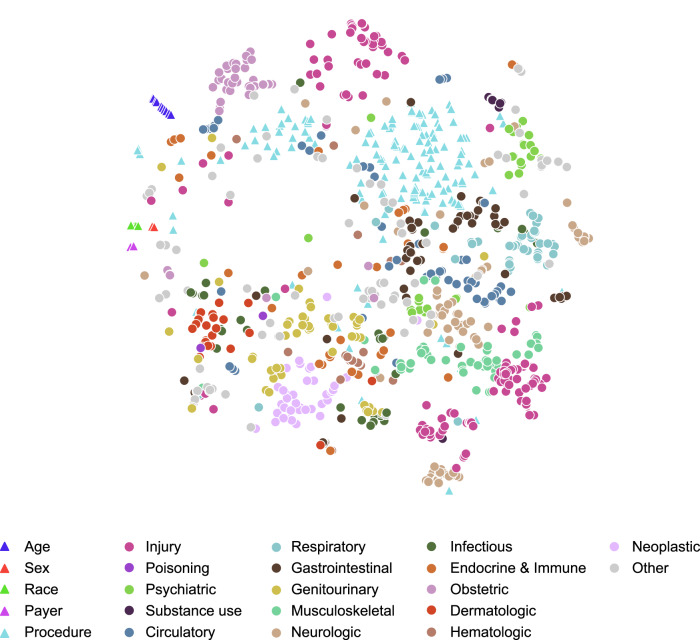
Clustering of pretrained code embeddings. Pretraining resulted in 780-dimensional embeddings for all 2174 features, including demographics, diagnosis and procedural codes, and temporal positions. This figure uses *t*-distributed stochastic neighbor embedding to depict the 1152 demographic, diagnostic, and procedural features with at least 1000 occurrences in the development dataset, with diagnoses colored by Clinical Classification Software categories. The proximity of two features is related to the similarity of their predictive significance within a patient trajectory. See interactive version at https://web.stanford.edu/~davidak/d3_tsne/index.html.

PABLO produced informative embeddings of visit features (Fig. 3), with clustering of empirically related diagnoses (e.g., injury and musculoskeletal, or psychiatric and neurologic diagnoses). Injury codes also displayed distinct clusters representing different types of traumas (e.g., falling, overdose toxicity, and bite wounds). Procedures often performed in conjunction with certain diagnoses were also clustered together (e.g., burn injuries and wound debridement).

### Trajectory analysis

**Fig. 4:**
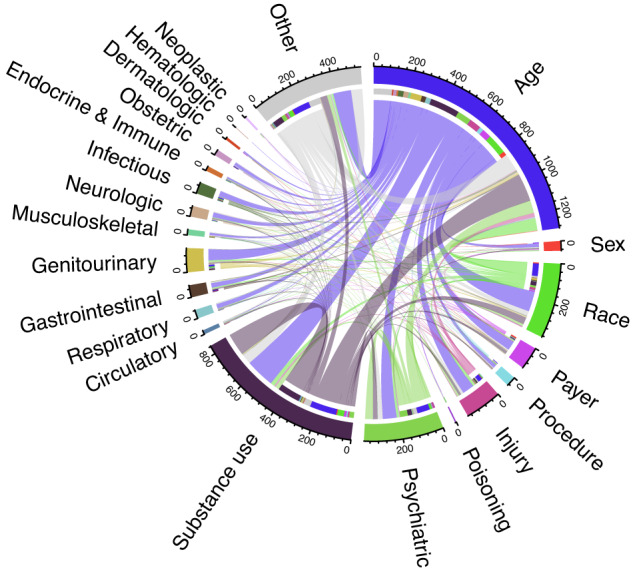
Most influential patient-level predictors of non-accidental trauma. This diagram displays the two most influential features for non-accidental trauma (NAT) prediction, determined by integrated gradient attributions, for all patient trajectories leading to NAT in the California test set. The flows between segments connect the two most highly weighted features for each trajectory, and are colored by the most influential feature. The proportion of the circumference occupied by each category is proportional to the likelihood of a feature in that category being the first or second most influential predictor of a patient’s NAT diagnosis.

**Fig. 5:**
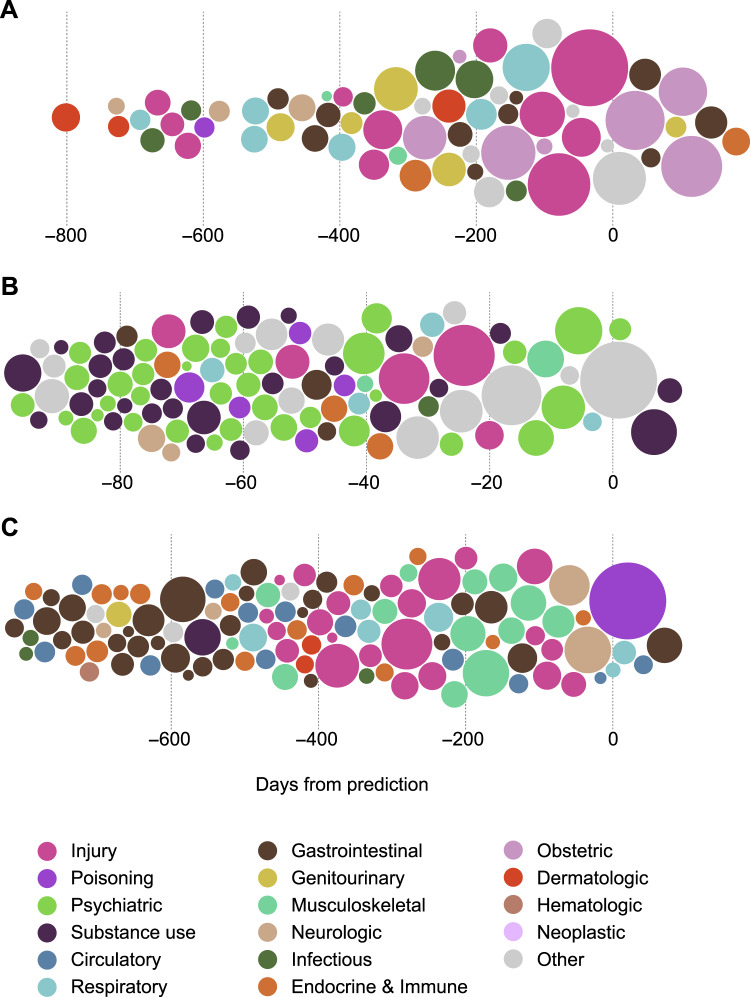
Patient trajectories leading to non-accidental trauma. Three patient trajectories representative of common non-accidental trauma (NAT) patterns, with details permuted to protect patient privacy. Circles represent diagnoses, ordered by days from the index visit and colored by diagnostic category. The area of each circle is linearly proportional to the magnitude of its integrated gradients attribution score, reflecting its influence on PABLO’s prediction of NAT. **A** A young pregnant patient with a history of traumatic diagnoses. **B** A homeless patient with a history of substance abuse and psychiatric diagnoses. **C** A patient with potential cases of previously misdiagnosed or undiagnosed NAT. See interactive version at https://web.stanford.edu/~davidak/d3_trajectory/index.html.

The most influential patient-level predictors of NAT are shown in Fig. 4. Substance use, psychiatric, and accidental injury diagnoses were important predictors of NAT, particularly in the context of specific age and racial categories. Visualizations of individual case trajectories (Fig. 5) demonstrate how model predictions and feature attributions can be integrated into a clinical decision support tool. This representation enables the identification of common patterns leading to NAT, included representative cases of pregnancy-associated abuse (Fig. 5A), homelessness, substance abuse, and psychiatric diagnoses (Fig. 5B), and a case with multiple apparently accidental injuries suggesting potentially undiagnosed NAT (Fig. 5C). Representative false positive trajectories (Supplementary Fig. 3) demonstrate attention to known risk factors, including prior trauma and substance use. Test characteristics in patient subgroups (Supplementary Tables 6–7) suggest higher false positive rates in Black patients and in patients with prior psychiatric, substance use, injury, and homelessness diagnoses. On the other hand, representative false negative trajectories (Supplementary Fig. 4) demonstrate difficulty in predicting NAT cases without classic risk factors, or whose trajectories contain less information. Higher false negative rates were observed in Asian patients and in patients without prior psychiatric, substance use, injury, or homelessness diagnoses (Supplementary Tables 6–7).

**Fig. 6:**
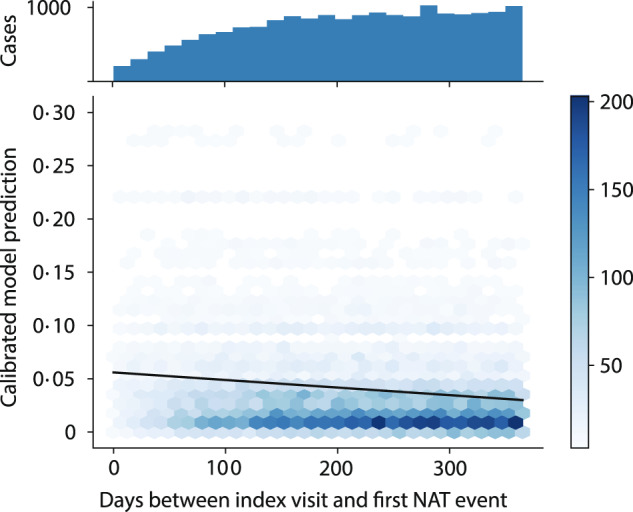
Predicted probability of NAT by lag between index visit and first NAT (FL external validation). Histogram shows the distribution of lag times between the index visit and first NAT event. Patient trajectories leading to NAT had a median lag of 216 (IQR 135-291) days between prediction and NAT event. Trendline shows linear regression of calibrated NAT prediction on prediction lag. For NAT cases, the mean calibrated predicted probability of NAT declined by 1% for each additional 100 days between prediction and event.

Patients experiencing NAT had a median lag between index visit and NAT of 218 days (IQR 136-291) in the CA test set and 216 days (IQR 135-291) in the FL external validation dataset. Prediction confidence declined with increasing lag between index visit and NAT, likely reflecting the fact that a longer delay between prediction and event implies more unobserved information influencing the event (and therefore weaker predictions). For NAT cases, the mean calibrated predicted probability of NAT declined by 1% for each additional 100 days between prediction and event (Fig. 6, Supplementary Fig. 5).

## Discussion

We developed and externally validated PABLO, a pretrained, longitudinal deep learning model for the prediction of NAT. To our knowledge, this is the largest study of NAT prediction to date, and the first to report external validation of the predictive model. The data used for prediction are ubiquitous in health systems, allowing for implementation in any setting collecting patient demographic, diagnostic, and procedural data.

PABLO significantly outperformed both traditional machine learning algorithms (i.e., logistic regression, XGBoost) and a state-of-the-art deep-learning model (BEHRT). These improvements may reflect our model’s attention to the temporal structure of patient trajectories, which is generally discarded in conventional algorithms using flattened representations of patient histories^23^. PABLO may also have benefited from a pretraining methodology that combines both contextual masked representation learning and multiple diagnosis forecasting tasks. Because risk stratification for NAT involves inferring the future course of a patient trajectory, this hybrid pretraining strategy may provide a more relevant foundation for our prediction task than typical masked token modeling alone. Indeed, pretraining substantially decreased the volume of training data required to attain a given level of performance.

Predicting NAT is complex, requiring identification of individuals at high risk of a rare and heterogenous outcome (NAT within one year) from an extremely large and diverse screening pool (all patients visiting any ED or hospital in the state). Therefore, while our model achieved high AUROCs, AUPRCs were comparatively lower. Low PPV is typical in the prediction of rare outcomes (e.g., suicide^24^). In this study, flagging the top 1% of patients by predicted NAT risk yields overall PPVs from 0.121–0.169, meaning that a minority of patients flagged as high risk would in fact experience observed NAT. This suggests that models like PABLO are best used as an automated primary screen to flag high-risk patients for detailed, secondary screening, rather than as a diagnostic test on its own. Notably, PABLO’s PPV significantly outperformed comparator models at the same risk threshold, detecting 4.3% to 98.4% more cases of actual NAT than alternative models. Given the morbidity and mortality of undiagnosed NAT, we view these gains as potentially clinically meaningful. PPV was substantially higher for patients with more visits on record, who both had more informative trajectories, and were more likely to experience NAT. Indeed, many previous studies of disease prognostication have restricted cohorts to patients with large numbers of visits (ranging from five^19^ to 25 visits^22^), discarding the majority of the patient population with relatively few visits. By contrast, we studied all patients with three or more visits, a more representative but more sparsely documented population. In practice, because the proportion of patients to approach for secondary screening will vary with the resources and goals of the implementing institution, we report test characteristics at various thresholds.

Prediction performance in external validation (FL) was very similar to that in the CA test set. High performance in external validation may result from the large volume of multi-hospital data we used during training, the generality of the pretraining tasks, and the ubiquitous character of the demographic, diagnostic, and procedural data on which our approach relies. Nonetheless, model calibration will be necessary to achieve appropriate probability estimates in any target population.

Interpretability is crucial to the clinical utility of a predictive model. Clinicians may have higher confidence in a model that corroborates their own knowledge of risk factors, and many of the most commonly used predictive tools (e.g., for myocardial infarction^25^ or pulmonary embolus^26^) consist of a small number of features reflecting major risk factors. We demonstrate that individual predictions from PABLO (a model with 26,977,920 parameters) can be explained using feature attributions, producing concise explanations of individual risk. These attributions reflect known risk factors for NAT. For instance, pregnancy and the postpartum period are associated with elevated risk for homicide^27^, which was reflected in the model’s attention to pregnancy-related diagnoses. Drug and alcohol use has also been correlated with adolescent homicides^28^, which was reflected in our model’s high attributions for substance use diagnoses, particularly for younger patients. Inspection of individual cases also revealed instances of the model omitting clinically relevant risk factors, such as repeated asphyxia and dyspnea diagnoses or non-assault injury diagnoses. These omissions were primarily observed in lower-risk demographic segments (e.g., elderly patients), indicating the importance of demographic context to model predictions. Review of false positive trajectories indicated an over-weighting of potentially irrelevant attributes, such as dermatologic conditions and idiopathic musculoskeletal pain. These patterns may relate to the representational adjacency of these diagnoses to true NAT risk factors in the model. We propose that concise, interactive visualizations of model predictions and attributions for individual patients will allow clinicians to rapidly assess the plausibility of a given prediction.

To facilitate longitudinal patient review and model interpretation, we developed an interactive web framework for visualizing patient medical trajectories and highlighting patient-specific risk factors, in this case for NAT, though the approach can be applied to any prediction task. We propose that the NAT prediction model could be applied as an automated, universal screen, with alert thresholds set by deploying institutions. Clinicians could then inspect model predictions and risk factors for flagged high-risk patients, thereby enabling tailored follow up questions, and where applicable, delivery of preventive interventions, such as urgent social work referral, psychiatric therapy, or substance abuse treatment.

Our study has limitations. Although we studied comprehensive statewide datasets with millions of patient trajectories, individual visits are summarized by a handful of diagnostic and procedural codes. A natural extension of our approach would be to include more granular, multi-modal, and in some cases unstructured data including clinical notes and imaging studies, which are not easily represented in conventional machine learning models, but are well-suited to transformer architectures^29,30^. We found a small number of disparities in model performance across patient subgroups. Lower predictive performance on younger patients and on Black patients may relate to higher rates of NAT in these populations, causing the model to overestimate individual risk. Conversely, Black patients may be at higher risk for undiagnosed NAT^31^, meaning that available datasets would incorrectly label some true cases as negative for NAT. Model biases are important factors to consider before deployment^32^, and mitigation methods such as model constraints^32^ and re-calibrations^33^ have been shown to reduce disparities in performance. Finally, we had access to data only from the United States, and cannot assess generalizability to other countries, which may have distinct profiles of risk for NAT, and vulnerable subgroups different from those in the United States.

We showed that PABLO, a pretrained, domain-adapted outcome forecasting model, can be used to predict both first and recurrent instances of NAT. PABLO maintained its performance in external validation and outperformed alternative models in multiple scenarios. Model behavior aligned with known risk factors for NAT, while making use of complex interactions and temporal dependencies to improve prediction accuracy. Future research will assess performance on additional diagnostic forecasting tasks, and prospective evaluation in clinical settings.

## Methods

### Data sources

The dataset used for model development and internal evaluation was obtained from California’s Department of Health Care Access and Information. We extracted all Emergency Department (ED) visit and inpatient admission records in the state of California (CA) from October 1, 2015 (the beginning of the ICD-10 coding period) to December 31, 2019, totaling 40,895,601 encounters from 446 facilities. The dataset used for external validation was obtained from the Agency for Healthcare Research and Quality, which included all ED visit and inpatient admission records in Florida (FL) from October 1, 2015 to December 31, 2019, totaling 31,771,462 encounters from 217 facilities.

### Trajectory construction and labeling

We built datasets around a single prediction point for each patient trajectory, which we refer to as the “index visit”: the patient’s last visit in 2018, so as to observe at least one year of data after the point of prediction. The pretraining dataset contained all patient trajectories from CA with two or more visits up to the index visit (for trajectories including at least one visit in 2018), or with two or more visits from 2015 to 2017 (for trajectories without visits in 2018). For pretraining, we used the category of the principal diagnosis from last visit as a multiclass label, with diagnosis categories defined by the Clinical Classification Software Refined (CCSR) for ICD-10-CM diagnoses^34^.

The fine-tuning and external validation datasets included all patient trajectories from CA and FL, respectively, with three or more visits, at least one occurring in 2018. We required at least three visits for fine-tuning and validation, compared to two visits for pretraining, so that fine-tuning and validation trajectories would retain longitudinal information (i.e., two or more visits) after removing the final visit during prediction. We defined NAT cases as trajectories including one or more NAT diagnoses within 365 days following the index visit (Fig. 2A). Diagnoses of NAT were adapted from the broader of Reis and colleagues’ two definitions^14^, and included the ICD-10 codes referenced in Supplementary Fig. 6. To evaluate the prediction of a patient’s first NAT diagnosis, we also created restricted testing and external validation datasets that excluded patients with NAT diagnoses prior to the index visit. The fine-tuning dataset was divided into a development (training and validation) dataset containing 90% of trajectories, and a held-out test set containing 10% of trajectories.

Demographic attributes were extracted from the index visit, including age (grouped by decade), sex, race, and primary visit payor. Each visit included ICD-10 codes for the principal diagnosis, up to 24 auxiliary diagnoses, and up to five external cause of injury codes, which classify injuries by mechanism and intent. ICD-10 codes were truncated to the first three characters, to group similar diagnoses. Visits also included Current Procedural Terminology (CPT) codes for procedures performed in the ED and ICD-10 codes for procedures associated with hospital admissions, both of which were translated into a common lexicon of 231 procedural categories using CCS for Services and Procedures^35^.

### Model development, pretraining, and finetuning

We developed PABLO as follows. All features of a patient trajectory were converted into dense embedding representations (Fig. 2B). Each diagnosis or procedure embedding consisted of a pooled summation of a code embedding, a code type embedding, and a temporal position embedding. The code embedding represented either a truncated ICD-10 code or CCS procedural code category. The code type embedding indicated whether it was a principal or auxiliary code, providing a relative priority between codes. The temporal position embedding represented the time interval in days between the visit and the index visit, grouped into seven categories: 0, 1–29, 30–89, 90–179, 180–359, 360–719, and 720+ days. We selected these categories to produce a roughly uniform distribution, and to reflect the intuition for a nonlinear relationship between time and prediction (i.e., that the difference between an event occurring 1 or 30 days from prediction is more meaningful than the difference between its occurrence 101 or 130 days from prediction). For downstream classification tasks, we used a CLS embedding to summarize the entire patient trajectory up to the index visit^21^. We fixed trajectories to 512 embedded features: longer trajectories were truncated to include information from only the more recent visits, and shorter trajectories were padded to a length of 512 using a padding token ignored in model computations. These trajectories, represented as 512 × *d* matrices, were then fed into a BERT encoder.

We pretrained PABLO using a multitask objective that included both MLM and next diagnosis prediction. For MLM, features were masked with a probability of 15%. Masked features were replaced with a MASK token with an 80% probability, randomly permuted with a 10% probability, or left unchanged with a 10% probability. The original values of the masked embeddings were predicted using a decoder. In addition to MLM, PABLO was pretrained to predict the clinical category of the next principal diagnosis of each pretraining trajectory, a task designed to reflect general risk stratification across all diagnosis categories in the pretraining dataset. Concretely, given a trajectory with *v* visits, all codes from the last visit *v* were masked, and the previous *v* - 1 visits were used to predict the clinical category of the principal diagnosis of visit *v* using a feed-forward layer over the pooled model output (Fig. 2C). For both tasks, we used cross-entropy loss functions, which were summed to generate a multi-task pretraining loss.

After pretraining, we fine-tuned PABLO for prediction of NAT using the pooled CLS embedding as input to a feed-forward layer and sigmoid function (Fig. 2D). We used a binary cross-entropy loss function in addition to loss weighting and weighted random sampling to address the low prevalence of NAT. We calibrated model predictions using isotonic regression^36^ (Supplementary Fig. 7). We evaluated model performance by AUROC. To account for the low prevalence of NAT, we also calculated AUPRC. We calculated additional performance metrics at thresholds determined by taking the top *k*% of predictions as positive cases (Supplementary Tables 1–3), as practical decision thresholds will depend on the local epidemiology and resources of model deployment sites. We computed 95% confidence intervals and significance tests for all performance metrics using bootstrapping with 10,000 resamples. Specifically, to estimate 95% CIs for differences in AUROC or AUPRC between models, we used the differences of the AUROC/AUPRC between models for each of 10,000 paired bootstrap resamples of the pertinent test set. That is, we calculated AUROC/AUPRC for PABLO and its comparison model on each of 10,000 replicates of the test set, then recorded the 2.5th and 97.5th percentiles of the empirical distribution of performance differences. Estimates were verified using parametric methods^37,38^.

For both pretraining and fine-tuning, we determined model hyperparameters via Bayesian optimization (Supplementary Table 8). We used the AdamW optimizer and linear decay with warmup as our learning rate schedule. We implemented PABLO using PyTorch (1.10.1) and adapted BERT from the HuggingFace Transformers^17^ library (4.21.2) with default settings where not otherwise specified. All training was conducted on an NVIDIA Tesla V100 GPU.

### Comparison models

As a simple baseline model, we used logistic regression fit on a set of known NAT risk factors^39–41^, including patient demographics and binary variables indicating whether the patient had any prior psychiatric, substance use, injury, pregnancy, or homelessness diagnoses. The full model and fitted coefficients are shown in Supplementary Table 9.

We compared our model to XGBoost^42^, a highly performant traditional machine learning algorithm using gradient-boosted decision trees. We trained XGBoost using one-hot encoded features, such that patient trajectories were represented as vectors of binary features, each indicating whether the patient received a particular diagnosis or procedure at any point up to the prediction visit. We likewise used Bayesian hyperparameter optimization to select the best performing XGBoost models (Supplementary Table 10).

We also compared our model to BEHRT, a state-of-the-art deep learning model for diagnosis prediction also based on BERT^19^. We implemented the model as described by Li and colleagues^19^ and pretrained BEHRT using MLM on our pretraining dataset for the same duration as PABLO. We then used Bayesian optimization to identify the optimal hyperparameter settings for BEHRT to predict NAT (Supplementary Table 11).

### Model interpretation and visualization

We used *t*-distributed stochastic neighbor embedding (*t*-SNE) to visualize the *d-*dimensional feature embeddings with over 1000 occurrences in the development dataset. To interpret model predictions, we used the integrated gradients technique^43^, which was chosen for its axiomatic properties and implementation efficiency. Unlike standard statistical models whose measures of feature importance are averaged across the population, transformers weight features based on contextual interactions. This contextual weighting allows for unique, patient-specific attributions for each feature. We calculated attribution scores for trajectories in the CA test dataset using the Captum library (0.6.0).

Using PABLO’s predictions and feature attributions, we developed an interactive clinical interface to visualize patient trajectories and risk factors. To preserve anonymity, we generated six representative true positive trajectories by identifying subpopulations of similar NAT cases in the CA test set and selecting representative examples from each subpopulation. 30% of features in each trajectory were then randomly resampled from their respective cohorts. We excluded from visualization diagnoses with fewer than 1000 occurrences. We generated analogous trajectories for false positive and false negative predictions, selecting the non-NAT cases with the highest predicted probabilities (i.e., false positives), and the NAT cases with the lowest predicted probabilities (i.e., false negatives).

### Software

Datasets were extracted and joined using R (4.0). Analyses were performed using Python (3.7.12). Data processing and cohort statistical analyses were performed using NumPy (1.21.6), pandas (1.3.5), and scikit-learn (0.24.2). Model development was performed using scikit-learn (0.24.2), XGBoost (1.5.0), PyTorch (1.10.1), Transformers (4.21.2), and Weights & Biases (0.13.2). Model analyses were performed using scikit-learn (0.24.2) and Captum (0.6.0). Visualizations were created using Matplotlib (3.4.2), R (4.0), and D3.js (7.8.2).

### Reporting summary

Further information on research design is available in the Nature Research Reporting Summary linked to this article.

## Supplementary information

Supplementary Information

Reporting Summary

## Data Availability

The specific data used in this study cannot be made public by the authors due to restrictions from California’s Department of Healthcare Access and Information (https://hcai.ca.gov/data-and-reports/research-data-request-information/) and the Agency for Healthcare Research and Quality (https://www.hcup-us.ahrq.gov/tech_assist/centdist.jsp). However, the datasets are available upon request to these organizations (links above).

## Code availability

Our code is publicly available at https://github.com/davidhhuang/PABLO.

## References

[1] Christoffel KK (1990). Violent death and injury in US children and adolescents. Am. J. Dis. Child..

[2] Rosenfeld EH (2020). Understanding non-accidental trauma in the United States: a national trauma databank study. J. Pediatr. Surg..

[3] Cheng D, Horon IL (2010). Intimate-partner homicide among pregnant and postpartum women. Obstet. Gynecol..

[4] Wallace ME, Friar N, Herwehe J, Theall KP (2020). Violence as a direct cause of and indirect contributor to maternal death. J. Women’s Health (Larchmt).

[5] Feldman KW (2001). The cause of infant and toddler subdural hemorrhage: a prospective study. Pediatrics.

[6] Singh GK, Azuine RE, Siahpush M, Kogan MD (2013). All-cause and cause-specific mortality among US youth: socioeconomic and rural-urban disparities and international patterns. J. Urban Health.

[7] Mercado MC, Holland K, Leemis RW, Stone DM, Wang J (2017). Trends in emergency department visits for nonfatal self-inflicted injuries among youth aged 10 to 24 years in the United States, 2001-2015. JAMA.

[8] Spong CY, Bianchi DW (2018). Improving public health requires inclusion of underrepresented populations in research. JAMA.

[9] Rhodes KV, Dichter ME, Smith KL (2018). Challenges and opportunities for studying routine screening for abuse. JAMA.

[10] Curry SJ (2018). Screening for intimate partner violence, elder abuse, and abuse of vulnerable adults: US preventive services task force final recommendation statement. JAMA.

[11] Kwan K, Wiebe D, Cerdá M, Goldman-Mellor S (2019). Repeat assault injury among adolescents utilizing emergency care: a statewide longitudinal study. J. Emerg. Med..

[12] Deans KJ, Thackeray J, Groner JI, Cooper JN, Minneci PC (2014). Risk factors for recurrent injuries in victims of suspected non-accidental trauma: a retrospective cohort study. BMC Pediatr..

[13] Zhao C, Starke M, Tompson JD, Sabharwal S (2020). Predictors for nonaccidental trauma in a child with a fracture-a national inpatient database study. J. Am. Acad. Orthop. Surg..

[14] Reis BY, Kohane IS, Mandl KD (2009). Longitudinal histories as predictors of future diagnoses of domestic abuse: modelling study. BMJ.

[15] Liu X (2019). A comparison of deep learning performance against health-care professionals in detecting diseases from medical imaging: a systematic review and meta-analysis. Lancet Digit Health.

[16] Vaswani, A. et al. Attention is all you need. In *Proc. 31st International Conference on Neural Information Processing Systems*, 6000–6010 (Curran Associates, 2017).

[17] Wolf, T. et al. Transformers: state-of-the-art natural language processing. In *Proc*. *of the 2020 conference on empirical methods in natural language processing: system demonstrations*, 38–45 (Association for Computational Linguistics, 2020).

[18] Dosovitskiy, A. et al. An image is worth 16x16 words: transformers for image recognition at scale. In *Proc**9th International Conference on Learning Representations* (2021).

[19] Li Y (2020). BEHRT: transformer for electronic health records. Sci. Rep..

[20] Rasmy L, Xiang Y, Xie Z, Tao C, Zhi D (2021). Med-BERT: pretrained contextualized embeddings on large-scale structured electronic health records for disease prediction. NPJ Digit. Med..

[21] Devlin, J., Chang, M.-W., Lee, K. & Toutanova, K. BERT: pre-training of deep bidirectional transformers for language understanding. In *Proc. of the 2019 Conference of the North American Chapter of the Association for Computational Linguistics*, 4171-4186 (Association for Computational Linguistics, 2019).

[22] Zhang Z, Yan C, Zhang X, Nyemba SL, Malin BA (2022). Forecasting the future clinical events of a patient through contrastive learning. J. Am. Med. Inf. Assoc..

[23] Rajkomar A (2018). Scalable and accurate deep learning with electronic health records. NPJ Digit. Med..

[24] Su C (2020). Machine learning for suicide risk prediction in children and adolescents with electronic health records. Transl. Psychiatry.

[25] Six AJ, Backus BE, Kelder JC (2008). Chest pain in the emergency room: value of the HEART score. Neth. Heart J..

[26] Wells PS (2001). Excluding pulmonary embolism at the bedside without diagnostic imaging: management of patients with suspected pulmonary embolism presenting to the emergency department by using a simple clinical model and d-dimer. Ann. Intern. Med..

[27] Wallace M, Gillispie-Bell V, Cruz K, Davis K, Vilda D (2021). Homicide during pregnancy and the postpartum period in the United States, 2018–2019. Obstet. Gynecol..

[28] Hohl BC (2017). Association of drug and alcohol use with adolescent firearm homicide at individual, family, and neighborhood levels. JAMA Intern. Med..

[29] Huang, K., Altosaar, J. & Ranganath, R. ClinicalBERT: modeling clinical notes and predicting hospital readmission. Preprint at: https://ui.adsabs.harvard.edu/abs/2019arXiv190405342H (2019).

[30] Zhang, S. et al. Large-scale domain-specific pretraining for biomedical vision-language processing. Preprint at: https://arxiv.org/pdf/2303.00915.pdf (2023).

[31] Baidoo L, Zakrison TL, Feldmeth G, Lindau ST, Tung EL (2021). Domestic violence police reporting and resources during the 2020 COVID-19 stay-at-home order in Chicago, Illinois. JAMA Netw. Open.

[32] Paulus JK, Kent DM (2020). Predictably unequal: understanding and addressing concerns that algorithmic clinical prediction may increase health disparities. NPJ Digit. Med..

[33] Thompson HM (2021). Bias and fairness assessment of a natural language processing opioid misuse classifier: detection and mitigation of electronic health record data disadvantages across racial subgroups. J. Am. Med. Inform. Assoc..

[34] Clinical Classifications Software Refined (CCSR) for ICD-10-CM Diagnoses. Healthcare Cost and Utilization Project (HCUP). December 2022. Agency for Healthcare Research and Quality. www.hcup-us.ahrq.gov/toolssoftware/ccsr/dxccsr.jsp. 21413206

[35] HCUP CCS-Services and Procedures. Healthcare Cost and Utilization Project (HCUP). May 2021. Agency for Healthcare Research and Quality. www.hcup-us.ahrq.gov/toolssoftware/ccs_svcsproc/ccssvcproc.jsp. 21413206

[36] Huang Y, Li W, Macheret F, Gabriel RA, Ohno-Machado L (2020). A tutorial on calibration measurements and calibration models for clinical prediction models. J. Am. Med Inf. Assoc..

[37] DeLong ER, DeLong DM, Clarke-Pearson DL (1988). Comparing the areas under two or more correlated receiver operating characteristic curves: a nonparametric approach. Biometrics.

[38] Clopper CJ, Pearson ES (1934). The use of confidence or fiducial limits illustrated in the case of the binomial. Biometrika.

[39] Kaufman E (2016). Recurrent violent injury: magnitude, risk factors, and opportunities for intervention from a statewide analysis. Am. J. Emerg. Med..

[40] Parreco J, Rattan R (2018). Machine learning models for prediction of reinjury after penetrating trauma. JAMA Surg..

[41] Albini PT, Zakhary B, Edwards SB, Coimbra R, Brenner ML (2023). Intimate partner violence and pregnancy: nationwide analysis of injury patterns and risk factors. J. Am. Coll. Surg..

[42] Chen, T. & Guestrin, C. XGBoost: a scalable tree boosting system. In *Proc. 22nd ACM SIGKDD International Conference on Knowledge Discovery and Data Mining*, 785–794 (Association for Computing Machinery, 2016).

[43] Sundararajan, M., Taly, A. & Yan, Q. Axiomatic attribution for deep networks. In *Proc. 34th International Conference on Machine Learning* - *Volume 70*, 3319–3328 (JMLR.org, 2017).

