# Supplementary material for: Development and external validation of a pretrained deep learning model for the prediction of non-accidental trauma
Source: NPJ Digit Med. 2023 Jul 19;6:131. doi: 10.1038/s41746-023-00875-y (PMC10356774; doi:10.1038/s41746-023-00875-y)
Supplement: Supplementary file 1 — Supplementary Information [file 41746_2023_875_MOESM1_ESM.pdf]

Supplementary Information: Development and External Validation of a Pretrained Deep Learning Model for the Prediction of Non-Accidental Trauma

Supplementary Figure 1. Receiver operating characteristic curves for prediction of NAT. 2
Supplementary Table 1. PABLO performance metrics at selected thresholds (CA test set)..3
Supplementary Table 2. PABLO performance metrics at selected thresholds (FL external validation set). 4
Supplementary Table 3. Comparison of performance metrics by model, for top 1% predictions. 5
Supplementary Table 4. Subgroup analysis of fine-tuned PABLO (CA test set)...6
Supplementary Table 5. Subgroup analysis of fine-tuned PABLO (FL external validation set). 7
Supplementary Figure 2. Effect of pretraining on prediction performance..8
Supplementary Figure 3. Representative false positive trajectories. 9
Supplementary Table 6. Prediction sensitivity and specificity by subgroup (CA test set)...10
Supplementary Table 7. Prediction sensitivity and specificity by subgroup (FL external validation set). 11
Supplementary Figure 4. Representative false negative trajectories. 12
Supplementary Figure 5. Predicted probability of NAT by lag between index visit and first NAT (CA test set). 13
Supplementary Figure 6. Word cloud of ICD-10 code descriptions for NAT..14
Supplementary Figure 7. Calibration of PABLO fine-tuned for NAT prediction using isotonic regression. 15
Supplementary Table 8. Optimal hyperparameter values for PABLO. 16
Supplementary Table 9. Logistic regression model for NAT..17
Supplementary Table 10. Optimal hyperparameter values for XGBoost..18
Supplementary Table 11. Optimal hyperparameter values for BEHRT. 19

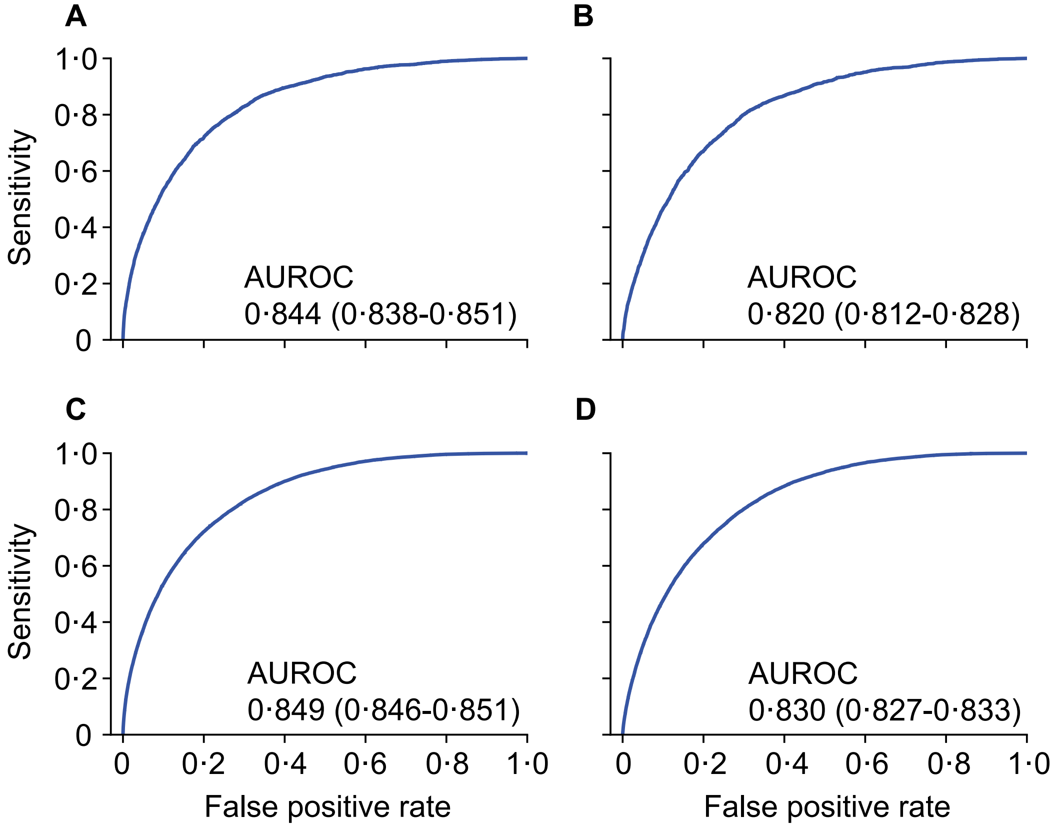

Supplementary Figure 1. Receiver operating characteristic curves for prediction of NAT.
(A) California test dataset (any NAT). **(B)** California test dataset restricted to patients who did not have previous NAT diagnoses (first NAT). **(C)** Florida external validation dataset (any NAT). **(D)** Florida external validation dataset restricted to patients who did not have previous NAT diagnoses (first NAT). AUROC=area under the receiver operating characteristic curve.

Supplementary Table 1. PABLO performance metrics at selected thresholds (CA test set).

	**Top 0.01%**	**Top 0.1%**	**Top 1%**	**Top 10%**
**Accuracy**	0.988 (0.988-0.988)	0.987 (0.987-0.988)	0.981 (0.981-0.982)	0.900 (0.899-0.902)
**Sensitivity**	0.003 (0.001-0.005)	0.027 (0.022-0.033)	0.138 (0.126-0.151)	0.520 (0.502-0.537)
**Specificity**	1.000 (1.000-1.000)	0.999 (0.999-0.999)	0.992 (0.991-0.992)	0.905 (0.904-0.906)
**PPV**	0.384 (0.200-0.577)	0.333 (0.275-0.392)	0.169 (0.155-0.182)	0.063 (0.061-0.065)
**NPV**	0.988 (0.988-0.988)	0.988 (0.988-0.988)	0.989 (0.989-0.990)	0.993 (0.993-0.994)
**FPR**	0.000 (0.000-0.000)	0.001 (0.001-0.001)	0.008 (0.008-0.009)	0.095 (0.094-0.096)
**FNR**	0.997 (0.995-0.999)	0.973 (0.967-0.978)	0.862 (0.849-0.874)	0.480 (0.463-0.498)
**FDR**	0.616 (0.423-0.800)	0.667 (0.608-0.725)	0.831 (0.818-0.845)	0.937 (0.935-0.939)
**F1 score**	0.006 (0.003-0.011)	0.051 (0.04-0.0610)	0.152 (0.140-0.165)	0.113 (0.109-0.117)

Values in parentheses are 95% confidence intervals. Each column represents model performance at a threshold determined by taking the top *k*% of predictions as positive cases. Therefore, these values represent performance in a clinical environment where resources are allocated to the top *k*% of patients at highest predicted risk for NAT. PPV=positive predictive value. NPV=negative predictive value. FPR=false positive rate. FNR=false negative rate. FDR=false discovery rate.

Supplementary Table 2. PABLO performance metrics at selected thresholds (FL external validation set).

	Top 0.01%	Top 0.1%	Top 1%	Top 10%
**Accuracy**	0.991 (0.991-0.991)	0.991 (0.991-0.991)	0.984 (0.984-0.984)	0.900 (0.900-0.901)
**Sensitivity**	0.004 (0.003-0.005)	0.029 (0.026-0.032)	0.143 (0.138-0.148)	0.527 (0.520-0.534)
**Specificity**	1.000 (1.000-1.000)	0.999 (0.999-0.999)	0.991 (0.991-0.991)	0.904 (0.903-0.904)
**PPV**	0.324 (0.261-0.386)	0.246 (0.228-0.265)	0.121 (0.117-0.126)	0.045 (0.044-0.045)
**NPV**	0.992 (0.992-0.992)	0.992 (0.992-0.992)	0.993 (0.993-0.993)	0.996 (0.995-0.996)
**FPR**	0.000 (0.000-0.000)	0.001 (0.001-0.001)	0.009 (0.009-0.009)	0.096 (0.096-0.097)
**FNR**	0.996 (0.995-0.997)	0.971 (0.968-0.974)	0.857 (0.852-0.862)	0.473 (0.466-0.480)
**FDR**	0.676 (0.614-0.739)	0.754 (0.735-0.772)	0.879 (0.874-0.883)	0.955 (0.955-0.956)
**F1 score**	0.008 (0.006-0.009)	0.052 (0.047-0.056)	0.131 (0.127-0.136)	0.083 (0.082-0.084)

Values in parentheses are 95% confidence intervals. Each column represents model performance at a threshold determined by taking the top *k*% of predictions as positive cases. Therefore, these values represent performance in a clinical environment where resources are allocated to the top *k*% of patients at highest predicted risk for NAT. PPV=positive predictive value. NPV=negative predictive value. FPR=false positive rate. FNR=false negative rate. FDR=false discovery rate.

Supplementary Table 3. Comparison of performance metrics by model, for top 1% predictions.

	Logistic regression	XGBoost	BEHRT	PABLO
**Accuracy**	0.983 (0.983-0.983)	0.984 (0.984-0.984)	0.984 (0.984-0.984)	0.984 (0.984-0.984) *
**Sensitivity**	0.071 (0.067-0.075)	0.133 (0.128-0.138)	0.136 (0.131-0.141)	0.143 (0.138-0.148) *†§
**Specificity**	0.991 (0.99-0.991)	0.991 (0.991-0.991)	0.991 (0.991-0.991)	0.991 (0.991-0.991)
**PPV**	0.061 (0.058-0.064)	0.113 (0.109-0.117)	0.116 (0.112-0.120)	0.121 (0.117-0.126) *†§
**NPV**	0.992 (0.992-0.992)	0.993 (0.993-0.993)	0.993 (0.993-0.993)	0.993 (0.993-0.993) *
**FPR**	0.009 (0.009-0.010)	0.009 (0.009-0.009)	0.009 (0.009-0.009)	0.009 (0.009-0.009)
**FNR**	0.929 (0.925-0.933)	0.867 (0.862-0.872)	0.864 (0.859-0.869)	0.857 (0.852-0.862) *†§
**FDR**	0.939 (0.936-0.942)	0.887 (0.883-0.891)	0.884 (0.880-0.888)	0.879 (0.874-0.883) *†§
**F1 score**	0.066 (0.062-0.069)	0.122 (0.118-0.127)	0.125 (0.120-0.129)	0.131 (0.127-0.136) *†§

Test characteristics represent performance in predicting any NAT in external validation, taking the top 1% highest predicted risk visits as positive cases. Ranges in parentheses are 95% confidence intervals. PPV=positive predictive value. NPV=negative predictive value. FPR=false positive rate. FNR=false negative rate. FDR=false discovery rate. * = $p < 0.05$ improvement over logistic regression, † = $p < 0.05$ improvement over XGBoost, § = $p < 0.05$ improvement over BEHRT. Confidence intervals and model comparisons calculated via bootstrapping with 10,000 resamples.

Supplementary Table 4. Subgroup analysis of fine-tuned PABLO (CA test set).

Population	Patients in sample	NAT Prev. (%)	Median visits (IQR)	Median no. facilities (IQR)	PABLO: AUROC (95% CI)	PABLO: AUPRC (95% CI)	XGBoost: AUROC (95% CI)	XGBoost: AUPRC (95% CI)
Sex								
Female	151,519	1.05	5 (4-8)	2 (1-3)	0.840 * (0.831-0.850)	8.77×10^{-2} * (7.76-10.06)	0.822 (0.813-0.832)	8.40×10^{-2} (7.34-9.66)
Male	105,890	1.47	5 (4-8)	2 (1-3)	0.845 * (0.836-0.853)	10.38×10^{-2} * (9.39-11.68)	0.829 (0.819-0.838)	9.58×10^{-2} (8.55-10.83)
Age								
<40	107,007	1.74	5 (4-8)	2 (1-3)	0.789 * (0.779-0.798)	8.93×10^{-2} * (8.01-10.09)	0.769 (0.759-0.779)	8.16×10^{-2} (7.33-9.26)
≥40	150,402	0.85	6 (4-9)	2 (1-3)	0.875 * (0.865-0.886)	10.43×10^{-2} * (9.28-11.83)	0.858 (0.847-0.869)	10.12×10^{-2} (8.89-11.62)
Race								
White	110,426	1.06	5 (4-8)	2 (1-3)	0.859 * (0.849-0.869)	8.69×10^{-2} * (7.67-10.0)	0.846 (0.835-0.856)	8.97×10^{-2} (7.79-10.42)
Black	34,563	2.14	6 (4-10)	2 (2-4)	0.793 * (0.777-0.809)	12.15×10^{-2} * (10.33-14.38)	0.770 (0.753-0.787)	10.41×10^{-2} (8.76-12.46)
Hispanic	92,588	1.22	5 (4-8)	2 (1-3)	0.835 * (0.823-0.847)	9.55×10^{-2} * (8.44-11.17)	0.814 (0.800-0.826)	8.80×10^{-2} (7.62-10.26)
Asian	15,522	0.48	5 (4-7)	2 (1-3)	0.871 * (0.828-0.905)	6.03×10^{-2} (3.14-11.27)	0.846 (0.798-0.884)	5.08×10^{-2} (2.91-10.61)
Native	1,383	1.37	6 (4-9)	2 (1-3)	0.743 * (0.647-0.834)	4.75×10^{-2} (2.50-15.58)	0.755 (0.661-0.846)	4.1×10^{-2} (2.56-9.41)
Other	2,927	0.51	4 (4-6)	1 (1-2)	0.786 (0.650-0.890)	2.86×10^{-2} (1.32-8.24)	0.795 (0.668-0.900)	3.15×10^{-2} (1.42-10.03)
Primary payor								
Medicaid	81,569	1.88	6 (4-9)	2 (1-3)	0.805 * (0.796-0.814)	10.96×10^{-2} * (9.93-12.17)	0.784 (0.773-0.793)	10.15×10^{-2} (9.15-11.38)
Medicare	103,513	0.52	6 (4-9)	2 (1-3)	0.876 * (0.857-0.895)	8.72×10^{-2} (6.96-11.37)	0.858 (0.837-0.878)	8.64×10^{-2} (6.71-11.13)
Self	54,743	1.98	5 (4-7)	2 (2-3)	0.777 * (0.747-0.805)	7.15×10^{-2} * (5.61-10.03)	0.766 (0.737-0.794)	6.69×10^{-2} (5.3-9.37)
Other	10,790	1.58	5 (4-8)	2 (2-3)	0.794 * (0.750-0.834)	7.53×10^{-2} * (5.17-12.45)	0.771 (0.725-0.816)	8.33×10^{-2} (4.92-13.36)
Private	6,794	0.80	5 (4-7)	2 (1-3)	0.828 * (0.809-0.845)	5.81×10^{-2} * (4.56-7.52)	0.803 (0.784-0.823)	6.03×10^{-2} (4.66-7.98)
Number of visits								
3-4	93,055	0.69	4 (3-4)	2 (1-2)	0.808 * (0.792-0.823)	2.99×10^{-2} * (2.62-3.67)	0.784 (0.768-0.799)	2.45×10^{-2} (2.17-2.86)
5-7	87,535	0.85	6 (5-6)	2 (1-3)	0.827 * (0.814-0.841)	4.40×10^{-2} * (3.89-5.17)	0.808 (0.794-0.822)	3.62×10^{-2} (3.22-4.20)
≥8	76,819	2.28	11 (9-15)	3 (2-4)	0.834 * (0.826-0.843)	13.43×10^{-2} * (12.31-14.79)	0.821 (0.811-0.830)	13.01×10^{-2} (11.82-14.41)

* $p < 0.05$ improvement compared to XGBoost (bootstrapping with 10,000 replicates).

Supplementary Table 5. Subgroup analysis of fine-tuned PABLO (FL external validation set).

Population	Patients in sample	NAT Prev. (%)	Median visits (IQR)	Median no. facilities (IQR)	PABLO: AUROC (95% CI)	PABLO: AUPRC (95% CI)	XGBoost: AUROC (95% CI)	XGBoost: AUPRC (95% CI)
Sex								
Female	1,306,784	0.84	6 (4-9)	3 (2-4)	0.848 * (0.845-0.851)	6.84×10^{-2} * (6.55-7.19)	0.830 (0.827-0.834)	6.34×10^{-2} (6.03-6.69)
Male	850,706	0.86	5 (4-8)	3 (2-4)	0.849 * (0.845-0.853)	6.72×10^{-2} * (6.35-7.17)	0.830 (0.826-0.834)	6.15×10^{-2} (5.79-6.57)
Age								
<40	950,487	1.25	6 (4-9)	3 (2-4)	0.794 * (0.790-0.797)	6.30×10^{-2} * (6.03-6.61)	0.772 (0.768-0.776)	5.80×10^{-2} (5.53-6.09)
≥40	1,207,001	0.54	5 (4-8)	3 (2-4)	0.884 * (0.881-0.888)	7.63×10^{-2} * (7.17-8.16)	0.863 (0.859-0.868)	7.07×10^{-2} (6.60-7.58)
Race								
White	1,142,171	0.78	5 (4-9)	3 (2-4)	0.859 * (0.856-0.863)	7.23×10^{-2} * (6.87-7.67)	0.842 (0.838-0.846)	6.51×10^{-2} (6.16-6.92)
Black	566,716	1.26	6 (4-9)	3 (2-4)	0.805 * (0.801-0.810)	6.74×10^{-2} * (6.38-7.15)	0.788 (0.783-0.793)	6.30×10^{-2} (5.94-6.71)
Hispanic	423,858	0.52	5 (4-8)	3 (2-4)	0.854 * (0.847-0.862)	5.43×10^{-2} (4.86-6.15)	0.830 (0.822-0.839)	5.17×10^{-2} (4.56-5.94)
Asian	11,707	0.32	5 (4-7)	2 (2-3)	0.900 * (0.846-0.944)	9.65×10^{-2} (4.36-20.59)	0.875 (0.817-0.927)	13.08×10^{-2} (4.61-24.91)
Native	2,168	1.29	6 (4-9)	3 (2-4)	0.862 (0.811-0.904)	6.78×10^{-2} (4.26-15.09)	0.836 (0.765-0.893)	5.93×10^{-2} (3.80-11.79)
Other	10,870	0.26	4 (4-6)	1 (1-2)	0.877 (0.794-0.944)	4.92×10^{-2} (2.16-14.13)	0.856 (0.778-0.92)	8.53×10^{-2} (1.71-20.21)
Primary payor								
Medicaid	685,479	1.18	6 (4-10)	3 (2-4)	0.811 * (0.806-0.816)	7.19×10^{-2} * (6.77-7.66)	0.792 (0.787-0.797)	6.57×10^{-2} (6.17-7.02)
Medicare	537,357	0.36	6 (4-8)	3 (2-4)	0.905 * (0.900-0.910)	6.69×10^{-2} (6.08-7.49)	0.879 (0.872-0.886)	6.70×10^{-2} (6.00-7.54)
Self	480,549	1.68	6 (4-9)	3 (2-4)	0.771 * (0.766-0.777)	7.39×10^{-2} * (6.94-7.92)	0.749 (0.743-0.755)	6.85×10^{-2} (6.42-7.34)
Other	328,502	1.24	6 (4-9)	3 (2-4)	0.829 * (0.819-0.838)	8.13×10^{-2} * (7.22-9.28)	0.809 (0.798-0.819)	7.31×10^{-2} (6.50-8.39)
Private	125,603	0.50	5 (4-7)	3 (2-4)	0.812 * (0.804-0.820)	3.37×10^{-2} * (2.98-3.91)	0.783 (0.773-0.792)	2.93×10^{-2} (2.60-3.43)
Number of visits								
3-4	740,244	0.47	4 (3-4)	2 (1-3)	0.825 * (0.819-0.831)	2.35×10^{-2} * (2.20-2.54)	0.798 (0.792-0.805)	1.93×10^{-2} (1.82-2.07)
5-7	731,561	0.55	6 (5-6)	2 (2-3)	0.829 * (0.823-0.834)	2.88×10^{-2} * (2.70-3.11)	0.807 (0.801-0.813)	2.50×10^{-2} (2.35-2.73)
≥8	685,685	1.59	11 (9-15)	3 (2-5)	0.831 * (0.828-0.834)	9.32×10^{-2} * (8.94-9.73)	0.814 (0.810-0.818)	8.68×10^{-2} (8.32-9.09)

* $p < 0.05$ improvement compared to XGBoost (bootstrapping with 10,000 replicates).

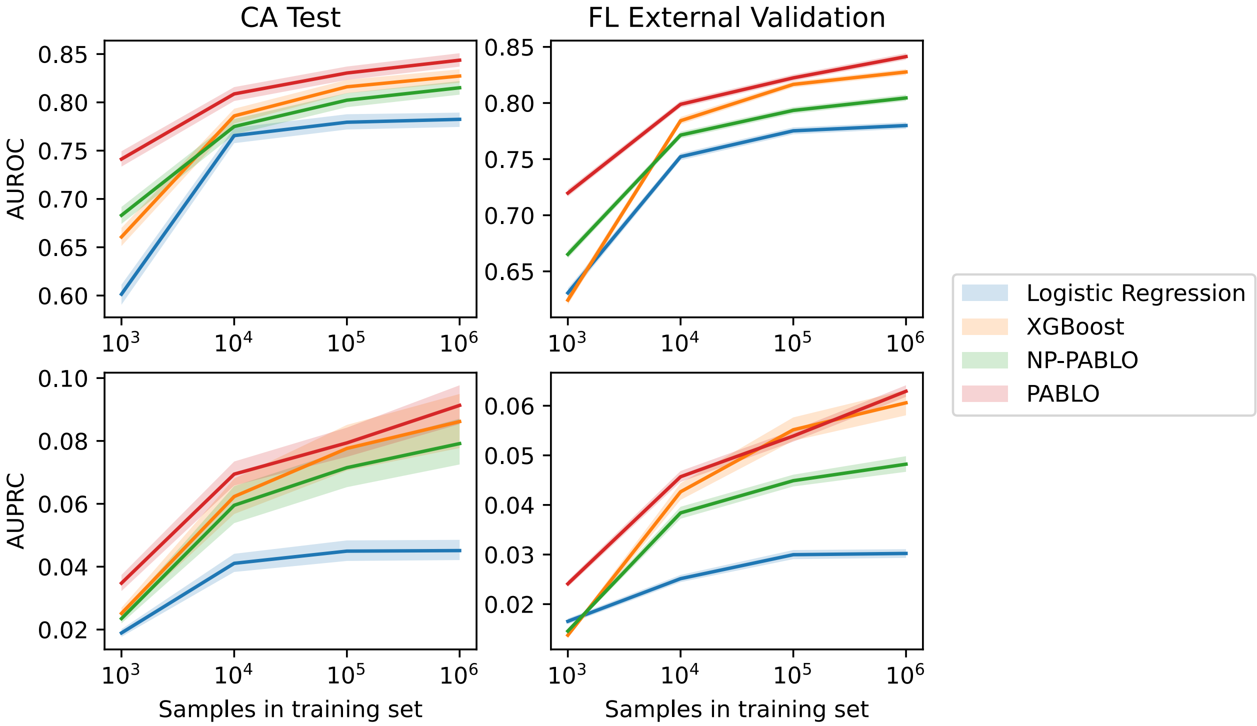

Supplementary Figure 2. Effect of pretraining on prediction performance.
Graphs compare performance of PABLO, non-pretrained PABLO (NP-PABLO), XGBoost, and logistic regression at various training set sizes. Shaded bands represent 95% confidence intervals, derived from bootstrap resampling with 10,000 replicates.

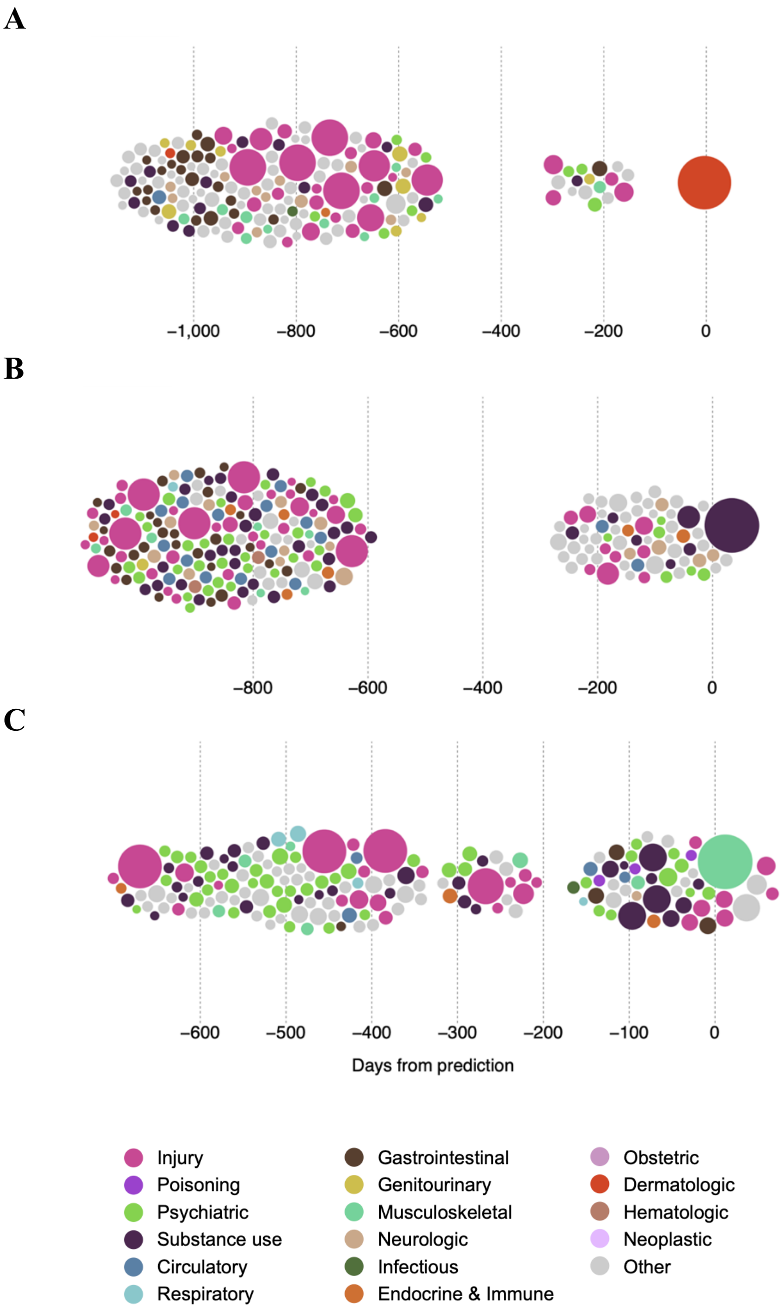

Supplementary Figure 3. Representative false positive trajectories.
(A) While the model is correctly attending to previous diagnoses of assault (pink), it is perhaps overweighting the importance of the rash diagnosis from the most recent visit (orange). **(B)** The model is correctly attending to previous assault diagnoses (pink) and an alcohol dependence diagnosis in the most recent visit (brown). Nonetheless, the patient did not receive an NAT diagnosis in the following year. **(C)** The model was correctly attending to previous assault (pink) and substance use diagnoses (brown). However, the model may be overweighting the importance of a musculoskeletal diagnosis for pain in the left wrist in the most recent visit (teal). This patient also had a history of suicidal ideation (green), which might justify this patient as high-risk for NAT.

Supplementary Table 6. Prediction sensitivity and specificity by subgroup (CA test set).

Population	Patients in sample	NAT prevalence (%)	Sensitivity	Specificity
Total			0.138 (0.126-0.151)	0.992 (0.991-0.992)
Sex				
Female	151,519	1.05	0.103 (0.088-0.118)	0.995 (0.995-0.996)
Male	105,890	1.47	0.175 (0.158-0.193)	0.986 (0.985-0.987)
Age				
<40	107,007	1.74	0.118 (0.104-0.133)	0.989 (0.989-0.99)
≥40	150,402	0.85	0.167 (0.148-0.188)	0.993 (0.993-0.994)
Race				
White	110,426	1.06	0.130 (0.111-0.15)	0.993 (0.992-0.993)
Black	34,563	2.14	0.207 (0.179-0.235)	0.977 (0.975-0.978)
Hispanic	92,588	1.22	0.111 (0.092-0.131)	0.994 (0.994-0.995)
Asian	15,522	0.48	0.041 (0.0-0.095)	0.999 (0.999-0.999)
Native	1,383	1.37	0.052 (0.0-0.158)	0.993 (0.988-0.997)
Other	2,927	0.51	0.0 (0.0-0.0)	1.0 (1.0-1.0)
Primary payor				
Medicaid	81,569	1.88	0.170 (0.154-0.187)	0.985 (0.984-0.986)
Medicare	103,513	0.52	0.133 (0.104-0.164)	0.997 (0.996-0.997)
Self	54,743	1.98	0.077 (0.044-0.112)	0.988 (0.986-0.99)
Other	10,790	1.58	0.098 (0.049-0.157)	0.988 (0.985-0.991)
Private	6,794	0.80	0.040 (0.024-0.058)	0.998 (0.997-0.998)
Number of visits				
3-4	93,055	0.69	0.003 (0.0-0.008)	1.0 (0.999-1.0)
5-7	87,535	0.85	0.019 (0.009-0.029)	0.998 (0.998-0.999)
≥8	76,819	2.28	0.238 (0.218-0.257)	0.974 (0.973-0.975)
Bottom income quartile				
No	199,991	1.11	0.131 (0.117-0.145)	0.992 (0.992-0.993)
Yes	57,418	1.61	0.155 (0.131-0.179)	0.989 (0.988-0.99)
Prior psychiatric diagnoses				
No	74,942	0.82	0.021 (0.011-0.034)	0.999 (0.999-0.999)
Yes	182,467	1.38	0.166 (0.153-0.181)	0.989 (0.988-0.989)
Prior substance use				
No	115,200	0.62	0.018 (0.01-0.028)	0.999 (0.999-0.999)
Yes	142,209	1.70	0.174 (0.158-0.189)	0.985 (0.985-0.986)
Prior injury				
No	53,558	0.65	0.0 (0.0-0.0)	1.0 (1.0-1.0)
Yes	203,851	1.37	0.155 (0.141-0.168)	0.989 (0.989-0.990)
Prior pregnancy				
No	133,490	1.21	0.079 (0.066-0.092)	0.993 (0.993-0.994)
Yes	123,919	1.23	0.201 (0.181-0.220)	0.990 (0.989-0.990)
Prior homelessness				
No	244,988	0.96	0.047 (0.039-0.055)	0.997 (0.997-0.998)
Yes	12,421	6.26	0.417 (0.382-0.450)	0.871 (0.865-0.877)

Operating point classifying the top 1% highest predicted risk patients as NAT cases.

Supplementary Table 7. Prediction sensitivity and specificity by subgroup (FL external validation set).

Population	Patients in sample	NAT prevalence (%)	Sensitivity	Specificity
Total			0.143 (0.138-0.148)	0.991 (0.991-0.991)
Sex				
Female	1,306,784	0.84	0.140 (0.134-0.147)	0.991 (0.991-0.992)
Male	850,706	0.86	0.146 (0.138-0.154)	0.991 (0.99-0.991)
Age				
<40	950,487	1.25	0.129 (0.123-0.135)	0.987 (0.987-0.988)
≥40	1,207,001	0.54	0.168 (0.16-0.177)	0.994 (0.994-0.994)
Race				
White	1,142,171	0.78	0.149 (0.141-0.156)	0.992 (0.992-0.992)
Black	566,716	1.26	0.153 (0.145-0.161)	0.984 (0.984-0.985)
Hispanic	423,858	0.52	0.091 (0.079-0.103)	0.997 (0.997-0.997)
Asian	11,707	0.32	0.0 (0.0-0.0)	1.000 (0.999-1.000)
Native	2,168	1.29	0.106 (0.0-0.214)	0.987 (0.983-0.992)
Other	10,870	0.26	0.0 (0.0-0.0)	1.000 (1.000-1.000)
Primary payor				
Medicaid	685,479	1.18	0.142 (0.133-0.15)	0.988 (0.987-0.988)
Medicare	537,357	0.36	0.154 (0.14-0.169)	0.996 (0.996-0.996)
Self	480,549	1.68	0.171 (0.16-0.181)	0.979 (0.978-0.979)
Other	328,502	1.24	0.191 (0.172-0.212)	0.983 (0.982-0.984)
Private	125,603	0.50	0.038 (0.031-0.047)	0.998 (0.998-0.999)
Number of visits				
3-4	740,244	0.47	0.008 (0.005-0.011)	1.0 (0.999-1.0)
5-7	731,561	0.55	0.022 (0.018-0.027)	0.999 (0.998-0.999)
≥8	685,685	1.59	0.230 (0.223-0.237)	0.974 (0.974-0.974)
Bottom income quartile				
No	1,540,151	0.76	0.140 (0.134-0.147)	0.992 (0.992-0.993)
Yes	617,339	1.09	0.147 (0.139-0.155)	0.988 (0.988-0.988)
Prior psychiatric diagnoses				
No	676,503	0.59	0.023 (0.018-0.028)	0.998 (0.998-0.999)
Yes	1,480,987	0.97	0.176 (0.170-0.182)	0.988 (0.988-0.988)
Prior substance use				
No	938,754	0.45	0.023 (0.019-0.028)	0.999 (0.999-0.999)
Yes	1,218,736	1.16	0.178 (0.172-0.185)	0.985 (0.985-0.985)
Prior injury				
No	428,855	0.44	0.001 (0.000-0.002)	1.000 (1.000-1.000)
Yes	1,728,635	0.95	0.159 (0.153-0.165)	0.989 (0.989-0.989)
Prior pregnancy				
No	1,146,621	0.80	0.083 (0.077-0.088)	0.995 (0.994-0.995)
Yes	1,010,869	0.91	0.202 (0.194-0.21)	0.987 (0.987-0.987)
Prior homelessness				
No	2,109,741	0.75	0.086 (0.082-0.09)	0.995 (0.995-0.995)
Yes	47,749	5.20	0.504 (0.486-0.522)	0.829 (0.825-0.832)

Operating point classifying the top 1% highest predicted risk patients as NAT cases.

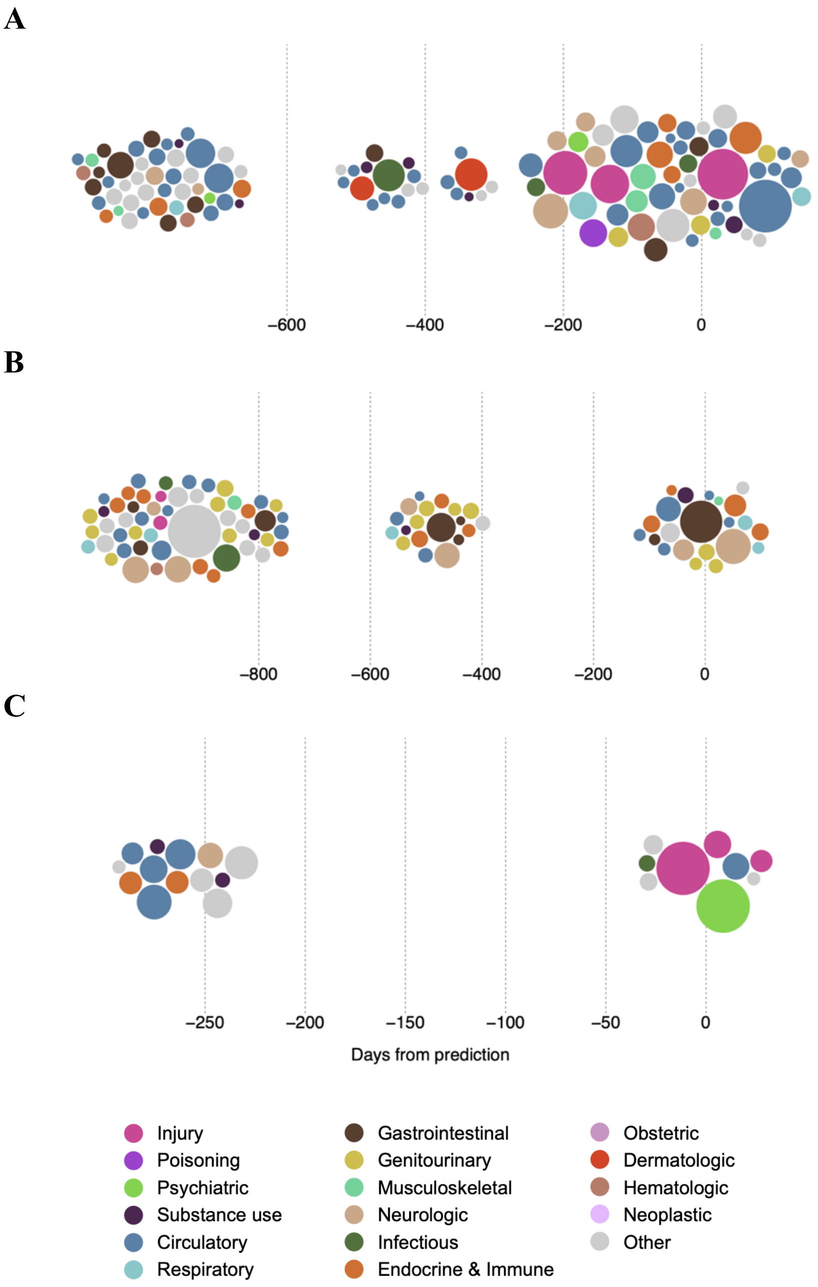

Supplementary Figure 4. Representative false negative trajectories.
(A) While this patient does not display conventional risk factors for NAT, this patient had non-assault injury diagnoses (pink) related to falling and choking that were perhaps overlooked by the model. The demographic characteristics of this patient (i.e., old age) may have also misled the model into categorizing this patient as low-risk for NAT. **(B)** This patient presents with no apparent risk factors for NAT, and was classified as low risk, but nevertheless experienced NAT. **(C)** This patient displays some NAT risk factors, such as recent injury diagnoses related to a hand laceration (pink) in the context of a psychiatric complaint (green), but was nevertheless misclassified as low-risk.

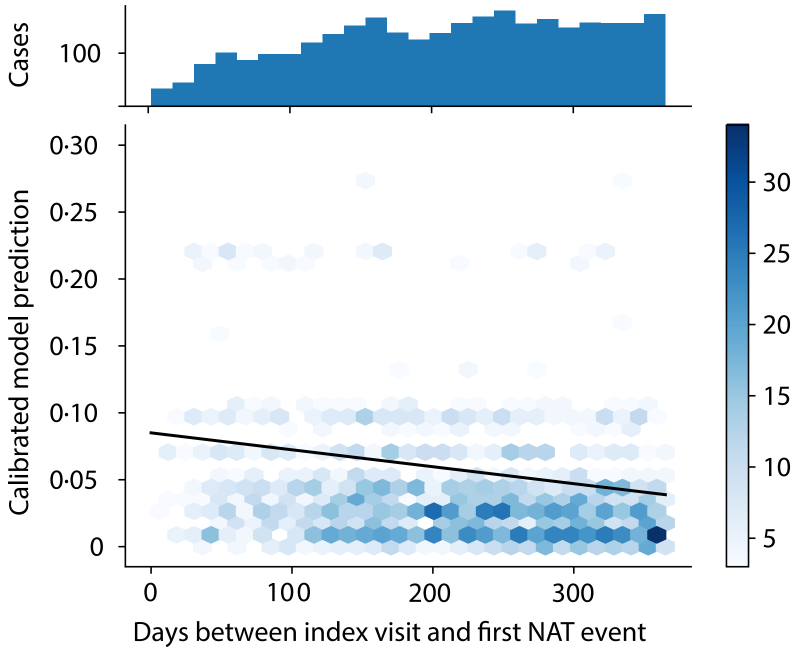

Supplementary Figure 5. Predicted probability of NAT by lag between index visit and first NAT (CA test set).
Histogram shows the distribution of lag times between the index visit and first NAT event. Patient trajectories leading to NAT had a median lag of 217.5 (IQR 136-291) days. Trendline shows linear regression of calibrated NAT prediction on prediction lag. For NAT cases, the mean calibrated predicted probability of NAT declined by 0.7% for each additional 100 days between prediction and event.

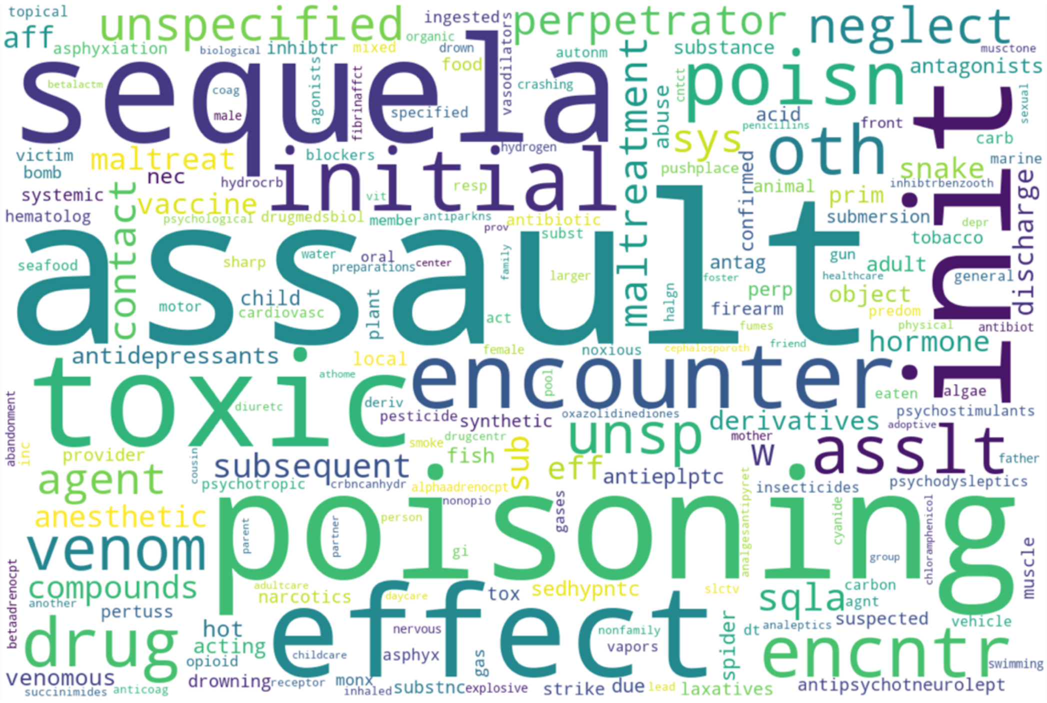

Supplementary Figure 6. Word cloud of ICD-10 code descriptions for NAT. Descriptions of the 1,389 ICD-10 codes for NAT are available at https://github.com/davidhhuang/PABLO.

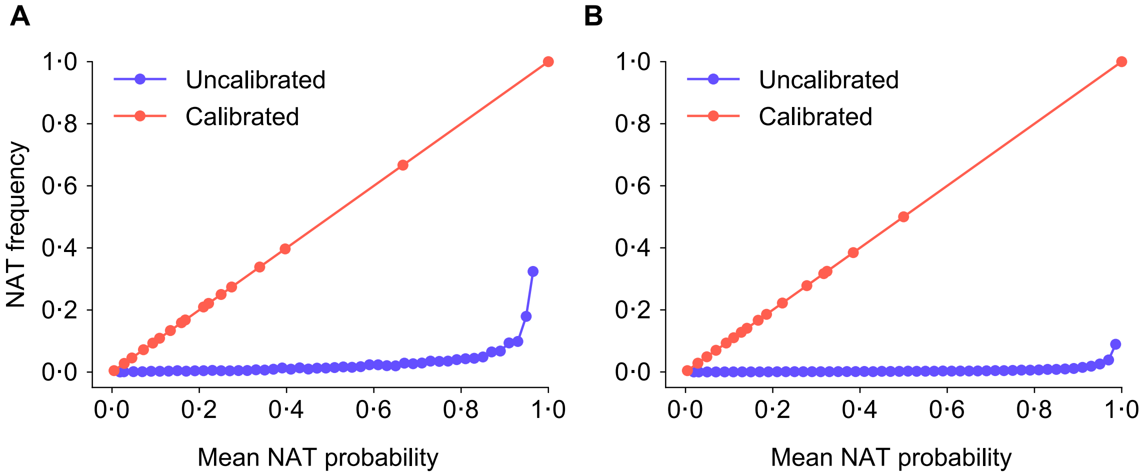

Supplementary Figure 7. Calibration of PABLO fine-tuned for NAT prediction using isotonic regression.
Calibration curves before and after application of isotonic regression on **(A)** the CA test set and **(B)** the FL external validation set.

Supplementary Table 8. Optimal hyperparameter values for PABLO.

**Hyperparameter**	**Pretraining value**	**Fine-tuning value**
adam_weight_decay	0.5	1e-3
attention_probs_dropout_prob	0.1	0.3
batch_size	32	32
classifier_dropout	0.1	0.2
epochs	5	1
hidden_dropout_prob	0.2	0.3
hidden_size	780	780*
intermediate_size	800	800*
learning_rate	4e-4	2e-5
num_accumulation_steps	7	1
num_attention_heads	3	3*
num_hidden_layers	6	6*
pos_weight	N/A	80
sample_weight	N/A	2
scheduler_warmup_steps	N/A	40

We used a Bayesian hyperparameter search with hyperband early stopping over 30 iterations for both pretraining and fine-tuning. *Value fixed after pretraining.

Supplementary Table 9. Logistic regression model for NAT.

	**Odds Ratio (95% CI)**
Intercept	0.003 (0.003-0.003)
Age: 0-9	0.222 (0.192-0.258)
Age: 10-19	1.722 (1.631-1.818)
Age: 20-29	1.523 (1.463-1.585)
Age: 30-39	1.235 (1.185-1.288)
Age: 50-59	0.705 (0.673-0.739)
Age: 60-69	0.39 (0.366-0.415)
Age: 70-79	0.164 (0.148-0.182)
Age: 80-89	0.099 (0.086-0.115)
Age: 90-99	0.088 (0.068-0.115)
Sex: Female	0.927 (0.903-0.952)
Race: Asian	0.651 (0.598-0.71)
Race: Black	1.604 (1.552-1.658)
Race: Hispanic	0.996 (0.965-1.027)
Race: Native	1.261 (1.091-1.457)
Race: Other	0.451 (0.364-0.559)
Payer: Medicaid	1.644 (1.584-1.707)
Payer: Medicare	1.512 (1.433-1.595)
Payer: Self	1.634 (1.539-1.734)
Payer: Other	1.596 (1.483-1.716)
Income: Bottom quartile	1.147 (1.115-1.18)
Prior: Psychiatric diagnoses	1.187 (1.144-1.232)
Prior: Substance use	1.941 (1.872-2.013)
Prior: Injury	1.856 (1.777-1.939)
Prior: Pregnancy	0.904 (0.879-0.929)
Prior: Homelessness	3.762 (3.643-3.885)

Non-accidental trauma (NAT) in the next year was regressed on previously reported risk factors for NAT. Sex=Male, Race=White, Payer=Private, and Age=40-49 not shown.

Supplementary Table 10. Optimal hyperparameter values for XGBoost.

**Hyperparameter**	**Value**
colsample_bytree	0.514
gamma	7.189
learning_rate	4.15e-2
max_depth	10
min_child_weight	100
reg_lambda	0.001
scale_pos_weight	62
subsample	0.5

We used a Bayesian hyperparameter search with 30 iterations.

Supplementary Table 11. Optimal hyperparameter values for BEHRT.

Hyperparameter	Pretraining value	Fine-tuning value
adam_weight_decay	0.1	3e-4
attention_probs_dropout_prob	0.1	0.3
batch_size	32	32
classifier_dropout	0.1	0.1
epochs	5	1
hidden_dropout_prob	0.2	0.3
hidden_size	540	540*
intermediate_size	820	820*
learning_rate	3e-4	3e-5
num_accumulation_steps	24	1
num_attention_heads	4	4*
num_hidden_layers	7	7*
pos_weight	N/A	42
sample_weight	N/A	51
scheduler_warmup_steps	N/A	91

We used a Bayesian hyperparameter search with hyperband early stopping to identify an optimal set of hyperparameter values for BEHRT during pretraining and fine-tuning. *Value fixed after pretraining.
