# Supplementary material for: Development and external validation of a pretrained deep learning model for the prediction of non-accidental trauma
Source: NPJ Digit Med. 2023 Jul 19;6:131. doi: 10.1038/s41746-023-00875-y (PMC10356774; doi:10.1038/s41746-023-00875-y)
Supplement: Supplementary file 2 — Reporting Summary [file 41746_2023_875_MOESM2_ESM.pdf]

nature portfolio

Corresponding author(s): David Kim MD PhD

Last updated by author(s): May 15, 2023

Reporting Summary

Nature Portfolio wishes to improve the reproducibility of the work that we publish. This form provides structure for consistency and transparency in reporting. For further information on Nature Portfolio policies, see our Editorial Policies and the Editorial Policy Checklist.

Statistics

For all statistical analyses, confirm that the following items are present in the figure legend, table legend, main text, or Methods section.

n/a	Confirmed	
☐	☒	The exact sample size (*n*) for each experimental group/condition, given as a discrete number and unit of measurement
☐	☒	A statement on whether measurements were taken from distinct samples or whether the same sample was measured repeatedly
☐	☒	The statistical test(s) used AND whether they are one- or two-sided<br>*Only common tests should be described solely by name; describe more complex techniques in the Methods section.*
☐	☒	A description of all covariates tested
☒	☐	A description of any assumptions or corrections, such as tests of normality and adjustment for multiple comparisons
☐	☒	A full description of the statistical parameters including central tendency (e.g. means) or other basic estimates (e.g. regression coefficient) AND variation (e.g. standard deviation) or associated estimates of uncertainty (e.g. confidence intervals)
☐	☒	For null hypothesis testing, the test statistic (e.g. *F*, *t*, *r*) with confidence intervals, effect sizes, degrees of freedom and *P* value noted<br>*Give P values as exact values whenever suitable.*
☒	☐	For Bayesian analysis, information on the choice of priors and Markov chain Monte Carlo settings
☒	☐	For hierarchical and complex designs, identification of the appropriate level for tests and full reporting of outcomes
☐	☒	Estimates of effect sizes (e.g. Cohen's *d*, Pearson's *r*), indicating how they were calculated

Our web collection on statistics for biologists contains articles on many of the points above.

Software and code

Policy information about availability of computer code

Data collection	N/A
Data analysis	Datasets were extracted and joined using R (4.0). Analyses were performed using Python (3.7.12). Data processing and cohort statistical analyses were performed using NumPy (1.21.6), pandas (1.3.5), and scikit-learn (0.24.2). Model development was performed using scikit-learn (0.24.2), XGBoost (1.5.0), PyTorch (1.10.1), Transformers (4.21.2), and Weights & Biases (0.13.2). Model analyses were performed using scikit-learn (0.24.2) and Captum (0.6.0). Visualizations were created using Matplotlib (3.4.2), R (4.0), and D3.js (7.8.2).

For manuscripts utilizing custom algorithms or software that are central to the research but not yet described in published literature, software must be made available to editors and reviewers. We strongly encourage code deposition in a community repository (e.g. GitHub). See the Nature Portfolio guidelines for submitting code & software for further information.

Data

Policy information about availability of data

All manuscripts must include a data availability statement. This statement should provide the following information, where applicable:

- Accession codes, unique identifiers, or web links for publicly available datasets
- A description of any restrictions on data availability
- For clinical datasets or third party data, please ensure that the statement adheres to our policy

The specific data used in this study cannot be made public by the authors due to restrictions from California’s Department of Healthcare Access and Information

(https://hcai.ca.gov/data-and-reports/research-data-request-information/) and the Agency for Healthcare Research and Quality (https://www.hcup-us.ahrq.gov/tech_assist/centdist.jsp). However, the datasets are available upon request to these organizations (links above).

Human research participants

Policy information about studies involving human research participants and Sex and Gender in Research.

Reporting on sex and gender	The underlying administrative datasets used report "sex", and we use the same reporting. We stratify analyses by sex (and other demographic characteristics) where relevant.
Population characteristics	Cohort characteristics are described in detail in Table 1.
Recruitment	We did not recruit participants. Data were derived from statewide administrative databases of ED visits and hospital admissions in California and Florida.
Ethics oversight	Stanford University Institutional Review Board

Note that full information on the approval of the study protocol must also be provided in the manuscript.

Field-specific reporting

Please select the one below that is the best fit for your research. If you are not sure, read the appropriate sections before making your selection.

☒ Life sciences ☐ Behavioural & social sciences ☐ Ecological, evolutionary & environmental sciences

For a reference copy of the document with all sections, see nature.com/documents/nr-reporting-summary-flat.pdf

Life sciences study design

All studies must disclose on these points even when the disclosure is negative.

Sample size	We studied all eligible patient trajectories in California and Florida databases of ED visits and hospital admissions, 2015-2019. The derivation of specific datasets and cohorts is described in Figure 1.
Data exclusions	We excluded from trajectory construction visits without a patient identifier, and patients with only a single visit in the data (precluding construction of a trajectory). Cohort construction is reported in detail in the Methods.
Replication	We have reproduced the main findings from the original data using the published code.
Randomization	N/A
Blinding	N/A, retrospective research on anonymized data.

Reporting for specific materials, systems and methods

We require information from authors about some types of materials, experimental systems and methods used in many studies. Here, indicate whether each material, system or method listed is relevant to your study. If you are not sure if a list item applies to your research, read the appropriate section before selecting a response.

Materials & experimental systems

n/a	Involved in the study	
☒	☐	Antibodies
☒	☐	Eukaryotic cell lines
☒	☐	Palaeontology and archaeology
☒	☐	Animals and other organisms
☒	☐	Clinical data
☒	☐	Dual use research of concern

Methods

n/a	Involved in the study	
☒	☐	ChIP-seq
☒	☐	Flow cytometry
☒	☐	MRI-based neuroimaging
